# The Foundress of St. Columba's

**Published:** 1920-10-02

**Authors:** 


### The Foundress of St. Columba's.

A very saintly and notable individual passed away on September 18 in the person of Miss Frances Mary Davidson. It has been given to few women to inaugurate and carry to so high a pitch of perfection a work so unique in its aims and character as Friedenheim, the Home of Peace, now known under the name of St. Columba's Hospital. Miss Davidson was the daughter of the late Mr. Patrick Davidson, of Inchmarlo, on Deeside. She was one of a large family, and was for many years in the habit of visiting the Mildmay Deaconesses' Home, where she found a strong impulse towards philanthropic work. It was in the year 1881 that her attention was first called to the great lack of any provision, save under the Poor-law, for patients at the point of death. After long and patient investigation into the means by which such sufferers could best be relieved, Miss Davidson gathered round her a group of sympathisers, and in 1885 opened the Home for the Dying, which for nearly forty years was her constant charge. The original Home contained but ten beds, and for the first experimental year was entirely maintained by its foundress. Another house was soon added and as soon outgrown. In 1892 the present hospital at Swiss Cottage was opened by the Duchess of Teck, who from the beginning had been greatly interested in Miss Davidson's aims. She was accompanied by the present Queen, and Her Majesty, who is now, with Princess Christian, patron of the hospital, recalled the circumstance when she saw Miss Davidson in Edinburgh some few weeks ago.

